# Phospholipase C gamma 1 (PLCG1) R707Q mutation is counterselected under targeted therapy in a patient with hepatic angiosarcoma

**DOI:** 10.18632/oncotarget.5503

**Published:** 2015-10-10

**Authors:** Hans Prenen, Dominiek Smeets, Massimiliano Mazzone, Diether Lambrechts, Xavier Sagaert, Raf Sciot, Maria Debiec-Rychter

**Affiliations:** ^1^ Digestive Oncology, University Hospitals Leuven and Department of Oncology, KU Leuven, Belgium; ^2^ Laboratory for Translational Genetics, Vesalius Research Center, Department of Oncology, KU Leuven, Leuven, Belgium; ^3^ Laboratory for Translational Genetics, Vesalius Research Center, VIB, Leuven, Belgium; ^4^ Laboratory of Molecular Oncology and Angiogenesis, Vesalius Research Center, Department of Oncology, KU Leuven, Leuven, Belgium; ^5^ Laboratory of Molecular Oncology and Angiogenesis, Vesalius Research Center, VIB, Leuven, Belgium; ^6^ Department of Pathology, KU Leuven and University Hospitals Leuven, Leuven, Belgium; ^7^ Department of Human Genetics, KU Leuven and University Hospitals Leuven, Leuven, Belgium

**Keywords:** angiosarcoma, sunitinib, *PLCG1* mutation, resistance, targeted therapy

## Abstract

Hepatic angiosarcoma is a rare and aggressive vascular neoplasm. Pathogenic driver mutations are largely unknown. We present the case of a patient with recurrent hepatic angiosarcoma, who initially showed good response to sunitinib, followed by progression. Using comprehensive molecular techniques, we explored the potential mechanisms of resistance. By low-read-depth whole-genome sequencing, the comparison of copy number aberrations (CNAs) of the primary tumor to the skin metastatic lesion that developed after progression on sunitinib, revealed high-level amplification of the 4q11-q13.1 region (containing *KIT, PDGFRA* and *VEGFR2* genes) that was sustained in both lesions. Whole exome sequencing on the germline, primary and metastatic tumor DNAs, resulted in 27 confirmed mutations, 19 of which (including *TP53* mutation) presented in both primary and metastatic lesions. One mutation, *ZNF331* frameshift deletion, was detected only in the primary tumor. In contrast, seven other mutations, including phospholipase C-gamma1 (*PLCG1)* R707Q mutation, were found only in the metastatic tumor, indicating selection of cells with the resistant genotype under sunitinib pressure. Our study supports the notion that *PLCG1*-R707Q mutation may confer VEGFR2-independent signaling and may thus cause resistance against VEGF(R)-directed therapies. This case illustrates also the advantages of using next-generation technologies in identifying individualized targeted therapy.

## INTRODUCTION

Primary hepatic angiosarcoma is a rare soft tissue tumor, accounting for only 2% of all liver malignancies [1]. This vascular tumor originates from endothelial cells from blood or lymphatic vessels and has an aggressive behavior. Radical surgery with R0 resection is the only curative treatment; however, the risk of recurrence remains high. Moreover, most patients are diagnosed with metastatic disease and this late diagnosis together with resistance against traditional chemo- and radiotherapy results in a poor outcome with a 2-year survival of only 3% of patients with metastatic disease [2]. Cytotoxic chemotherapy used for the treatment of metastatic disease includes anthracyclines, ifosfamide and taxanes, although there is only little evidence of activity in angiosarcoma. In most centers, taxanes are the treatment of choice for hepatic angiosarcoma given its anti-angiogenic properties [3, 4], even though retrospective analysis suggests that cutaneous angiosarcoma of the head and neck respond better to taxanes than angiosarcomas in other anatomical localizations, such as the liver [5]. Because angiosarcomas are malignant endothelial cell tumors, angiogenesis is believed to play an important role in its pathogenesis. Immunohistochemical analysis demonstrates overexpression of all the three subtypes of vascular endothelial growth factor receptors (VEGFR) [6], as well as the VEGF regulators, HIF-1α and β [7]. In about 10% of angiosarcomas, mutations in the VEGFR2 or VEGFR3 can be detected [8, 9], and gene expression profiling shows upregulation of *TIE1, VEGFR2, SNRK, TEK (TIE2)* and *FLT1 (VEGFR1)*, all playing a role in angiogenesis. In contrast with other sarcomas, angiosarcomas show a low level of alterations in p53 (only 4% mutations) and PIK3CA/AKT/mTOR pathways [10]. A recent study analyzed 39 angiosarcomas by whole-genome, whole-exome and targeted sequencing and revealed recurrent mutations in *PTPRB* and *PLCG1*, both linked with angiogenesis [11]. Furthermore, 38% of tumors displayed at least one driver mutation in angiogenesis signaling genes. In addition, *PLCG1*-R707Q missense mutation has been confirmed as a recurrent oncogenic event in angiosarcomas in another study [12].

Based on these molecular findings, several groups have explored the potential of anti-angiogenic agents such as the anti-VEGF monoclonal antibody bevacizumab, and the multi tyrosine kinase inhibitors sorafenib, sunitinib and pazopanib, with moderate results [13–16].

In this study, we present to our knowledge the first case of a hepatic angiosarcoma effectively treated with sunitinib. Moreover, we performed molecular analysis both pre- and post-treatment specimens of the tumor tissue to explore potential mechanisms of resistance.

## RESULTS

### Case presentation

**Figure 1:**
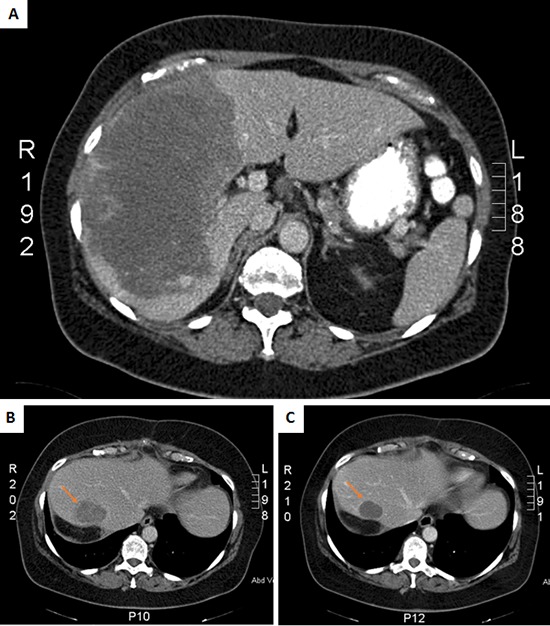
CT scans of primary tumor at diagnosis **A.** and the recurrent liver tumor at baseline **B.** and after 2 months treatment with sunitinib **C.**

A 56 year-old female was admitted in the emergency department with acute abdominal pain in the right hypochondriac region. Blood examination showed leukocytosis (17.33 × 10^9^/L), anemia (11 g/dL), elevated alkaline phosphatases (669 U/L), gamma-glutamyl transferases (117 U/L) and C-reactive protein (77 mg/dL). Both abdominal ultrasound and computer tomography (CT) revealed a hypervascular large liver mass in segment 5, 6, 7 and 8, with active bleeding (Figure 1A). An emergent celiac angiogram was conducted and showed contrast extravasation indicating an active tumor bleeding. Embolization was performed after super-selective cannulation until flow stasis. Two weeks later surgery was performed by laparoscopic right hemi-hepatectomy to remove the tumor. Both, frozen and formalin-fixed, paraffin embedded (FFPE) tissues from the resection specimen was available for molecular analysis.

Three months post-surgery, imaging by CT scan revealed a large local recurrence in the residual liver segment 4, as well as peritoneal implants, retroperitoneal lymph nodes and surrenal and lung metastases. A few days after the diagnosis of recurrence, the patient presented in the emergence department with an acute high gastro-intestinal bleeding, due to a duodenal metastasis of the angiosarcoma, for which she first received endoscopic treatment and subsequently embolization. Thereafter, sunitinib was administered to the patient at a dose of 37.5 mg daily. No significant side effects occurred, including fatigue, hand-food syndrome or gastrointestinal symptoms. Imaging after treatment with sunitinib for two months revealed a good response, with a decrease in tumor volume as well as a decrease in density (Figure 1B and 1C). After four months of treatment imaging by CT scan showed stable disease of the intra-abdominal lesions but progression of the lung metastases. Since the patient did not experience major side effects, the dose of sunitinib was increased to 50 mg daily continuously. The patient developed hypertension for which an antihypertensive drug was prescribed. Two months after the sunitinib dose increase evaluation by CT scan revealed progressive disease of both the abdominal as the lung metastases. Moreover, patient developed skin metastases, from which a tumor biopsy was taken again for molecular analysis. Second line therapy was planned, however before the start of this treatment, the patient developed left hemiplegia with secondary hemorragia due to brain metastases, and she died shortly after.

### Histopathology

**Figure 2:**
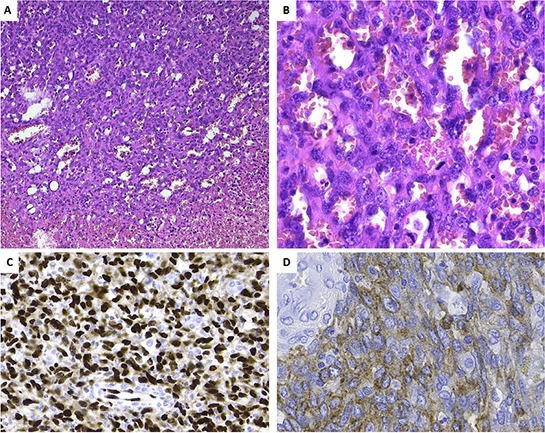
Histopathology and immunoprofile of the primary tumor **A.** Low power view, showing the transition from viable tumor to necrotic tumor. Note the presence of vascular channels. **B.** At high power the vascular channels are delineated by plump cells with atypical nuclei. Numerous mitoses are seen (arrows). Tumor cells are strongly positive for ERG **C.** and weakly for CD117 **D.**

Pathologic examination confirmed an infiltrative tumor composed of vascular channels delineated by atypical endothelial cells expressing the vascular markers CD31 and ERG, consistent with the diagnosis of angiosarcoma (Figure 2A and 2B). There was necrosis and mitotic figures were numerous. The tumor cells weakly expressed CD117 (Figure 2C).

### Cytogenetic and molecular analysis

**Figure 3:**
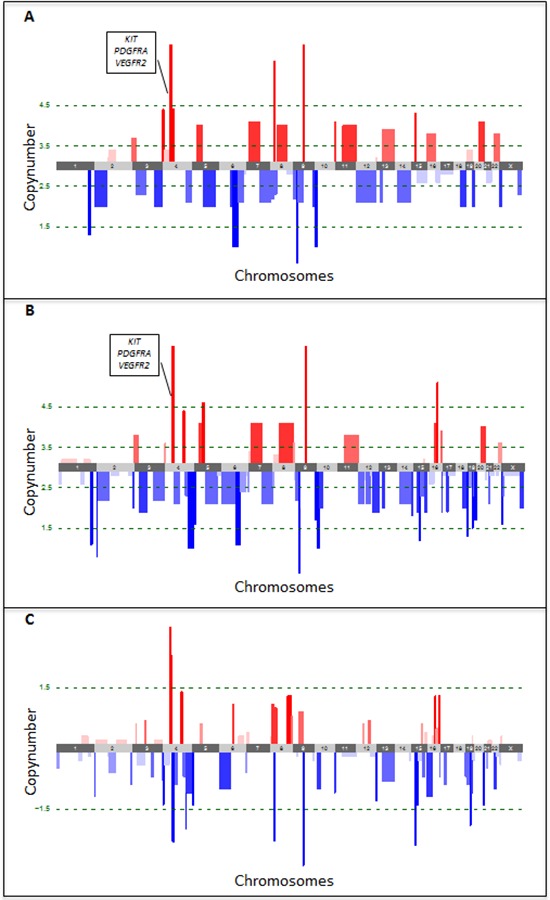
Copy number aberrations detected by low-read-depth whole-genome sequencing in the primary **A.** and metastatic **B.** tumor lesions. Amplifications are shown in red, deletions in blue. Green dotted lines represent the thresholds for classification of the CNAs in 5 categories based on their copy number (CN), i.e. high-level deletions (CN < 1.5) and amplifications (CN > 4.5), low-level deletions (1.5 < CN < 2.5) and amplifications (3.5 < CN < 4.5) and copy-neutral regions (2.5 < CN < 3.5). Copy number changes between both lesions were determined by subtracting the copy number of the primary from the metastatic tumor lesion **C.** Loss of copies is indicated in blue, gain of copies in red. The green dotted line represents the threshold for differences in copy number by more than 1.5.

The comparison of copy number aberrations (CNAs) of the primary tumor lesion to the skin metastatic lesion that developed after progression on sunitinib was performed by low-read-depth whole-genome sequencing using DNA isolated from FFPE tissues. After segmentation with ASCAT, both primary and metastatic lesions were found to be triploid. Subsequently CNAs were classified in 5 categories based on their copy number (CN) (see methods). In the primary tumor, 3 high-level amplifications and 4 high-level deletions were detected (Figure 3A, Supplementary Table S1). Notably, one of the high level amplifications involved the 4q11-q13.1 region, which contains *KIT, PDGFRA* and *VEGFR2*. In the metastatic lesion, 4 high-level amplifications and 8 high-level deletions were detected (Figure 3B, Supplementary Table S1). Although we also identified 8 regions that had a difference in copy number greater than 1.5 copies when comparing primary and metastatic tumor cell populations (Figure 3C, Supplementary Table S1), the amplification of the 4q11-q13.1 region was sustained in both lesions. Notably, high-level amplification of either *C-MYC*/8q24 or *FLT4*/5q35 was not present in both lesions. A list of the 1435 genes occurring in the 19 regions with either high-level amplifications or deletions is available upon request.

**Figure 4:**
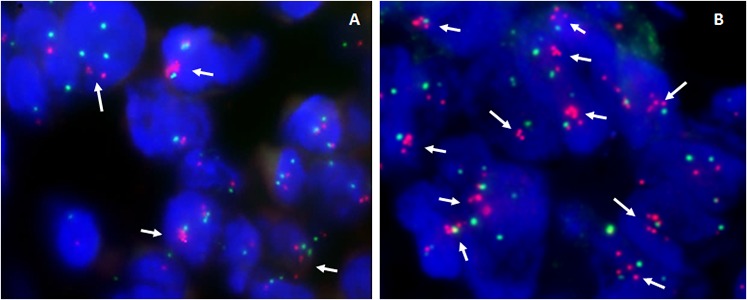
Representative examples of dual-color interphase FISH images on paraffin sections **A.** Primary liver angiosarcoma, showing amplification of *PDGFRA* and *KIT* genes (which maps at 4q12) in 33% of cells (arrows), as detected by the co-hybridization of SpectrumOrange labeled BAC's *PDGFRA*/RP11–24O10 and *KIT*/RP11–959G16 DNA probes (red signals) and SpectrumGreen labeled chromosome 4 CEP probe (green signals). **B.** Skin metastases of relapsed tumor - using the same combination of probes, amplification of *PDGFRA* and *KIT* genes in reference to chromosome 4 CEP probe is detected in majority of cells (>80%).

Fluorescence *In Situ* Hybridization (FISH) was carried out for the validation of the genomic copy number changes and evaluation of tumor chromosome ploidy level (Supplementary Table S2). Generally, genomic copy number changes detected by low-read-depth whole-genome sequencing were in line with results obtained by FISH. Of note, both lesions disclosed the presence of cells with the amplification of *KIT, PDGFRA, VEGFR2*/4q12 genes in reference to chromosome 4 centromeric probe, albeit significantly lower in number in primary *versus* metastatic lesion (33% *vs*. 81%, respectively) (Figure 4A and 4B), which was in good concordance with the tumor percentage of the biopsies by low-read-depth whole genome sequencing (17% *vs*. 48%, respectively).

**Figure 5:**
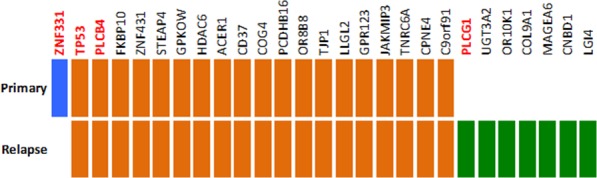
Somatic mutations detected by whole-exome sequencing and validated using ultra-deep targeted resequencing Primary-unique mutations are marked in blue and metastasis-unique mutations in green while mutations detected in both primary and metastasis are marked in orange.

Furthermore, we performed whole exome sequencing on the germline, primary tumor and metastatic tumor DNA, to determine somatic mutations. The detected mutations were validated using ultra-deep targeted resequencing, resulting in 27 confirmed mutations (Figure 5, Supplementary Table S3). Nineteen of those, including a mutation in *TP53*, were detected in both, primary and metastatic lesions. One mutation, namely a frameshift deletion in *ZNF331* was detected only in the primary tumor. In contrast, seven other non-synonymous or frameshift mutations (in genes *UGT3A2, PLCG1, OR10K1, COL9A1, MAGEA6, CNBD1* and *LG14*) were detected only in the metastatic tumor, indicating a selection and proliferative advantage of cells with the diverse genotype under sunitinib treatment.

## DISCUSSION

Angiosarcomas are generally resistant to chemotherapy and represent a tumor subset where novel therapeutic approaches are needed. In the patient described here, a good primary response had been seen after the administration of sunitinib, followed by the wide-spread metastatic refractory disease that developed six months later. Since underlying mechanisms of response and/or resistance to targeted therapy in this deadly but rare disease were never investigated, we aimed to explore underlying genomic aberrations and viable treatment options. To this end we have compared CNAs and somatic mutations in the primary tumor and the skin metastatic lesion that developed after progression on sunitinib, using respectively low-read-depth whole-genome and whole-exome sequencing.

Both, the primary and sunitinib-refractory tumor cells, harbored numerous and genome-wide distributed CNAs, with only partially overlapped regions of high-level gains/amplifications and/or losses. Importantly, primary lesion disclosed the presence of cells sub-clone (~30% of cells) with the amplification of the 4q12 region, encompassing receptor kinase *KIT, PDGFRA*, and *VEGFR2* genes. Recurrent gains of a small region of chromosome 4q12 containing *KIT* and *VEGFR2* genes have been reported in sporadic angiosarcomas [12], and overexpression of vascular endothelial growth factors and their receptors have been linked with the good response of these tumors to sunitinib [17]. Given the absence of activating missense mutations that confer sunitinib sensitivity, the amplification of *KIT, PDGFRA*, and/or *VEGFR2* may have represented the critical sunitinib target in our case and may explain the primary good response of the tumor to the drug. Nevertheless, the sub-clone with the 4q12 amplification has also uniformly dominated (~ 80% of cells) in the sunitinib-refractory metastatic lesion, indicating the selection of cells with the secondary molecular defect(s) accountable for sunitinib resistance. By whole-genome exome sequencing, 20 of non-synonymous or frameshift gene mutations were detected in sunitinib-naïve, primary tumor; all but one (*ZNF331* frameshift deletion) were present also in sunitinib-resistant, metastatic lesion. In contrast, seven mutations were found only in the metastatic tumor, including *PLCG1*-R707Q missense mutation. Previous reports have identified the latter as a recurrent somatic mutation in 9–30% of angiosarcomas [11, 12]. Further studies proved this mutation to cause constitutive activation of PLCG1 independently of receptor tyrosine kinases, and to enhance the aggressiveness of HUVEC endothelial cells *in vitro* by the increase of apoptosis resistance and upsurge of cells migration and invasiveness [12]. The *PLCG1*-R707Q mutation did not represent an initiating oncogenic event in our case. However, given the short time of the development of refractory disease, most likely this mutation pre-existed in some primary tumor cells and was selected as the aggressive metastatic sub-clone under the sunitinib pressure. This strongly supports the notion that the R707Q mutation impose the resistance to sunitinib, and thus might possibly cause resistance against other VEGF/VEGFR2-directed therapies. Alternatively, due to the high molecular genomic complexity of our case, the alternative mechanisms could play a role. The clinical usefulness of *PLCG1* mutational status as a possible predictive biomarker in angiosarcomas in the context of vascular-specific receptor tyrosine kinases directed therapies warrants further study. Finally, our approach illustrates the advantages of using next generation sequencing for molecular characterization of solid tumors.

## MATERIALs AND METHODS

### Histopathology

Histopathologic examination was performed on formalin fixed paraffin-embedded (FFPE) tissue. Five-micrometer sections were used for routine hematoxylin and eosin (H&E) and immunohistochemical stainings (avidin-biotin-peroxidase complex method), using the following antibodies: ERG (Abcam, monoclonal, clone EPR3864, prediluted), CD31 (DAKO, monoclonal, clone JC70A, prediluted), CD117 (DAKO, polyclonal, prediluted).

### Copy number alteration (CNA) detection by low-read-depth whole-genome sequencing

Shot-gun whole genome libraries were prepared using KAPA library preparation kit (KAPA Biosystems) according to the manufacturer's instructions. After quantification with qPCR, the resulting libraries were sequenced on a HiSeq2000 (Illumina) at low coverage generating 21, 125, 380 50 bp reads on average. Raw sequencing reads were mapped to the human reference genome (NCBI37/hg19) using Burrows-Wheeler Aligner (BWA v0.5.8a). Picard (v1.43) was used to remove PCR duplicates. CNAs were identified by binning the reads in 30Kb windows, correcting for genomic waves using the PennCNV software package [18] and the resulting number of reads per 30 Kb window were transformed into log R-values. The ASCAT algorithm version 2.0.1 [19] was used to segment the raw data and estimate tumor percentages and overall ploidy. Subsequently CNAs were classified in 5 categories based on their copy number (CN), i.e. high-level deletions (CN < 1.5) and amplifications (CN > 4.5), low-level deletions (1.5 < CN < 2.5) and amplifications (3.5 < CN < 4.5), and copy-neutral regions (2.5 < CN < 3.5).

### Fluorescence in situ hybridization (FISH)

For the validation of genomic copy number changes and evaluation of tumor chromosome ploidy level, either a commercially available centromere enumeration probes (CEP) or dual-color locus specific identifier (LSI) probes (all from Abbott Laboratories, Des Plaines, IL, USA), or the bacterial artificial chromosome (BAC) DNA probes differentially labeled with SpectrumGreen (SG) or SpectrumOrange (SO) (Abbott Molecular, Des Pllaines, IL, USA) were utilized for dual-color interphase FISH on the 5-μm paraffin tumor sections. The probes applied are listed in Supplementary Table S2. The BAC clones were selected based on their location in the UCSC Human Genome Browser Gateway (http://genome.ucsc.edu/cgi-bin/hgGateway,GRCh37/hg19), and were obtained from the BACPAC Resource Center (http://bacpac.chori.org). DNA isolation, probe labeling and hybridization were performed as previously described [20]. For analysis, 100 nuclei were scored manually for each set of probes in at least two different areas of the section using an Axioplan 2 (Jena, Germany) fluorescence microscope, and analyzed with CYTOVISION software. For CEP probes, from 0 to 1 signals per nucleus in >60% of cells was defined as a whole chromosome loss. For LSI probes, the ratio of red to green signals was calculated. Heterozygous or homozygous loss was delineated if ratio was <0.6 and <0.3, respectively. Amplification was defined by the ratio >2.0.

### Whole-exome sequencing

Shot-gun whole genome libraries were prepared using KAPA library preparation kit (KAPA Biosystems), according to the manufacturer's instructions. Whole exome enrichment was performed using the SeqCapV3 exome enrichment kit (Roche), following the manufacturer's instructions. Using the resulting whole-exome libraries 2 × 100 bp paired end reads were generated on a HiSeq2000 using a V3 flowcell resulting in an average coverage of 51.5x.

Raw sequencing reads were mapped to the human reference genome (NCBI37/hg19) using Burrows-Wheeler Aligner (BWA v0.5.8a) [21]. Picard (v1.43) was used to remove PCR duplicates [22]. The Genome Analysis Tool Kit (GATK) was used for local realignment around insertions and deletions, base recalibration, and single nucleotide variant calling [23]. Insertions and deletions were called using Dindel [24]. Somatic mutations were identified by subtracting variants and indels detected in the matched germ-line DNA from those found in the tumor DNA. Low quality mutations were removed based on mapping quality and coverage and subsequently ANNOVAR [25] was used to annotate the remaining mutations. Several databases were used to remove common variants (MAF > 1%) (dbSNP version 132, 1000 Genomes Project, Axiom Genotype Data Set and the Complete Genomics diversity panel (46 Hapmap individuals). For downstream analysis, exonic non-synonymous mutations and frame shift indels were selected.

### Ultra-deep targeted resequencing

Targeted sequencing primers for the detected mutations were designed using the MassARRAY Assay Design software. Universal sequence tags were added to the forward and reverse primers and the resulting oligos were used to generate amplicons (Roche FastStart High Fidelity PCR kit). In a second round of PCR, universal sequencing adapters containing a 10 base pair (bp) index were added to the target amplicons using Acces Array Barcode primers for Illumina Sequencing. Subsequently, the samples were denatured and sequenced on an Illumina MiSeq in a 2 × 75 bp paired end sequencing run using a V3 flowcell. CASAVA was used to generate and demultiplex fastq files. The raw sequencing reads were mapped to the human reference genome (build37) using BWA (v0.5.8a). Read counts of both variant and reference alleles were called using GATK and manually checked using IGV (average coverage 6393x)

### Data accessibility

The whole-exome and low-read-depth whole-genome sequencing data have been deposited at the EBI EGA under accession number EGAS00001001281.

## SUPPLEMENTARY TABLES




