# Supplementary material for: Phospholipase C gamma 1 (PLCG1) R707Q mutation is counterselected under targeted therapy in a patient with hepatic angiosarcoma
Source: Oncotarget. 2015 Oct 10;6(34):36418–25. doi: 10.18632/oncotarget.5503 (PMC4742186; doi:10.18632/oncotarget.5503)
Supplement: Supplementary file 1 [file oncotarget-06-36418-s001.pdf]

SUPPLEMENTARY TABLES

Supplementary Table S1: Copy number alterations (CNAs) detected using low-read-depth whole-genome sequencing. Chromosomal segments and their copynumber (CN) are given for the primary and metastatic tumor lesions. CNAs were classified in 5 categories based on their copy number (CN); i.e., high-level deletions (CN <1.5) and amplifications (CN >4.5), low-level deletions (1.5 < CN <2.5) and amplifications (3.5< CN <4.5) and copy-neutral regions (2.5< CN <3.5). Interesting regions were defined as regions with high-level amplification or deletions or regions with a difference in copy number between primary and metastasis of more than 1.5. For each of these 19 regions the cancer consensus genes are given.

Supplementary Table S2: Validation of genomic copy number changes of primary and metastatic tumor cells by FISH

FISH nr.	Tumor specimen	Gene (chromosome location)/ Centromer probe	Copy number range	% of abnormal	Interpretation
1	Primary	*PDGFRA+KIT*(4q12)-SO/CEP4-SG	3–7/2–3	33%	4q12 amplification
	Metastatic		3–8/2	81%	4q12 amplification
2	Primary	*EGFR* (7p12)-SO/CEP7-SG	3–4/3–4	19%	*EGFR*/CEP7 gain
	Metastatic		3–4/3–4	70%	*EGFR*/CEP7 gain
3	Primary	*MYC*(8q24)-SO/CEP8-SG	1–2/2–3	18%	*MYC* loss/CEP8 gain
	Metastatic		3–4/3–4	59%	*MYC*/CEP8 gain
4	Primary	*CDKN2A*(9p21)-SO/CEP9-SG	0–1/2	18%	*CDKN2A* loss
	Metastatic		0–1/2	68%	*CDKN2A* loss
5	Primary	*PTEN*(10q23)-SO/CEP10-SG	2/2	0%	Diploid
	Metastatic		2/2	0%	Diploid

Abbreviations: CEP - centromeric enumeration probe, SO-SpectrumOrange, SG-SpectrumGreen

Supplementary Table S3: Mutations detected by whole-exome sequencing and validated using ultra-deep targeted resequencing

hg 19 Genomic position	Gene	Type	Allele change	Amino Acid change	Allele frequency germ-line	Total reads germ-line	Allele frequency primary tumor	Total reads primary tumor	Allele frequency metastasis	Total reads metastasis
chr19:54080634:54080635	ZNF331	frameshift deletion	820_821del	274_274del	0%	405	11%	202	0%	63
chr17:39974399:39974399	FKBP10	nonsynonymous SNV	G450T	W150C	0%	8269	8%	8629	20%	10508
chr17:7578475:7578475	TP53	nonsynonymous SNV	C59T	P20L	0%	2936	16%	802	62%	213
chr20:9417666:9417666	PLCB4	frameshift insertion	2595_2596insC	V865fs	0%	8104	8%	9014	19%	11021
chr19:21366268:21366268	ZNF431	nonsynonymous SNV	A1162G	T388A	0%	310	20%	575	56%	205
chr7:87911981:87911981	STEAP4	nonsynonymous SNV	G959T	R320I	0%	1969	14%	4071	42%	942
chrX:48972614:48972614	GPKOW	nonsynonymous SNV	A977C	Q326P	0%	5850	9%	2286	29%	1704
chrX:48682137:48682137	HDAC6	nonsynonymous SNV	G3245C	G1082A	0%	10975	7%	10711	18%	10028
chr19:6312434:6312434	ACER1	nonsynonymous SNV	G170A	R57H	0%	7596	7%	9473	24%	5594
chr19:49842627:49842627	CD37	nonsynonymous SNV	T725G	L242R	0%	7352	16%	7627	67%	5532
chr16:70551548:70551548	COG4	nonsynonymous SNV	G350A	R117H	0%	1727	10%	1615	27%	397
chr5:140563476:140563476	PCDHB16	nonsynonymous SNV	G1342A	A448T	0%	8247	11%	7990	33%	5749
chr11:124310617:124310617	OR8B8	nonsynonymous SNV	G365A	R122H	0%	11988	16%	11161	47%	12402
chr15:30010598:30010598	TJP1	stopgain SNV	C3748T	Q1250X	0%	8725	17%	10054	71%	12970
chr17:73565207:73565207	LLGL2	nonsynonymous SNV	C1471T	R491C	0%	6618	8%	6177	26%	7490
chr10:134942261:134942261	GPR123	nonsynonymous SNV	G929A	R310H	0%	7707	16%	8142	36%	2436
chr10:133930830:133930830	JAKMIP3	nonsynonymous SNV	G385A	E129K	0%	9651	8%	9534	11%	11002
chr16:24828160:24828160	TNRC6A	nonsynonymous SNV	A4855G	K1619E	0%	12184	8%	10990	27%	14122
chr3:131418773:131418773	CPNE4	nonsynonymous SNV	G412T	A138S	0%	7494	9%	7614	22%	1764
chr9:117379485:117379485	C9orf91	nonsynonymous SNV	G10A	E4K	0%	6800	7%	8397	23%	8924
chr5:36049073:36049073	UGT3A2	nonsynonymous SNV	C659A	A220D	0%	6937	0%	8342	15%	8272
chr20:39795235:39795235	PLCG1	nonsynonymous SNV	G2120A	R707Q	0%	6465	0%	7460	17%	6098
chr1:158436238:158436238	OR10K1	nonsynonymous SNV	A887G	E296G	0%	759	0%	2802	60%	370

(*Continued*)

hg 19 Genomic position	Gene	Type	Allele change	Amino Acid change	Allele frequency germ-line	Total reads germ-line	Allele frequency primary tumor	Total reads primary tumor	Allele frequency metastasis	Total reads metastasis
chr6:70993492:70993492	COL9A1	nonsynonymous SNV	A728T	D243V	0%	2226	0%	1481	37%	350
chrX:151869849:151869849	MAGEA6	nonsynonymous SNV	C539A	T180N	0%	7530	0%	7859	26%	8555
chr8:87917418:87917418	CNBD1	frameshift insertion	268_269insA	Q90fs	0%	4995	0%	7720	11%	4703
chr19:35622364:35622364	LGI4	nonsynonymous SNV	G554A	C185Y	0%	10983	0%	9981	25%	10911

For each of the mutations the genomic position, gene, type of mutation and change of nucleic or amino acid are given as well as the total number of reads and allelic frequencies in the germ-line, primary and metastasis sample
